# Impact of mobile application and outpatient follow-up on renal endpoints and physiological indices in patients with chronic kidney disease: a retrospective cohort study in Southwest China

**DOI:** 10.1186/s12911-024-02567-3

**Published:** 2024-06-12

**Authors:** Yu Shi, Shi Pu, Hongmei Peng, Jing Zhang, Yang Li, Xia Huang, Caiping Song, Yu Luo

**Affiliations:** 1 Department of Nephrology, The Second Affiliated Hospital, Army Medical University (Third Military Medical University), Chongqing, 400037 P.R. China; 2 Thinmed Medical Technology (Chongqing) Co.,LTD, Chongqing, 401121 P.R. China; 3 President Office, The Second Affiliated Hospital of Army Medical University (Third Military Medical University), Chongqing, 400037 P.R. China; 4 School of Nursing, Army Medical University, Third Military Medical University, No. 30 Gaotanyan Street, Shapingba District, Chongqing, 400038 P.R. China

**Keywords:** Mobile app, Chronic kidney disease, Remote follow-up, Outpatient follow-up, Retrospective cohort study

## Abstract

**Background:**

Chronic kidney disease (CKD) is a significant public health concern, and patient self-management is an effective approach to manage the condition. Mobile applications have been used as tools to assist in improving patient self-management, but their effectiveness in long-term outpatient follow-up management of patients with CKD remains to be validated. This study aimed to investigate whether using a mobile application combined with traditional outpatient follow-up can improve health outcomes of patients with CKD .

**Methods:**

This retrospective cohort study recruited CKD patients with stage 1–5 who were not receiving renal replacement therapy from a CKD management center. Two groups were established: the APP + outpatient follow-up group and the traditional outpatient follow-up group. Baseline data was collected from January 2015 to December 2019, followed by a three-year long-term follow-up until December 2022. Laboratory data, all-cause mortality, and renal replacement treatment were then collected and compared between the two groups.

**Results:**

5326 patients were included in the study, including 2492 in the APP + outpatient group and 2834 in the traditional outpatient group. After IPTW virtualization matching, the final matched the APP + outpatient group consisted of 2489 cases (IQR, 33–55) and 2850 (IQR, 33–55) in the traditional outpatient group. By the end of the study, it was observed that the laboratory data of Phosphorus, Sodium, Triglyceride, Hemoglobin showed significant improvements, Furthermore the APP + outpatient group demonstrated superior results compared to the traditional outpatient group (*P* < .05). And it was observed that there were 34 deaths (1.4%) in the APP + outpatient group and 46 deaths (1.6%) in the traditional outpatient group(*P* = .49). After matching for renal replacement therapy outcomes, the two groups were found to be comparable (95% CI [0.72–1.08], *P* = .23), with no significant difference. However, it was noted that the traditional outpatient group had a lower incidence of using temporary catheters during initial hemodialysis (95% CI [8.4-29.8%], *P* < .001).

**Conclusion:**

The development and application of an app combined with outpatient follow-up management can improve patient health outcomes. However, to ensure optimal preparation for kidney replacement therapy, patients in CKD stages 4–5 may require more frequent traditional outpatient follow-ups, and further develop an information-based decision-making support tool for renal replacement therapy.

**Supplementary Information:**

The online version contains supplementary material available at 10.1186/s12911-024-02567-3.

## Introduction

Chronic Kidney Disease (CKD) is a prevalent and costly health condition worldwide. The prevalence of CKD is 10.8% in China and as high as 18.3% in the southwest regions [1]. As CKD progresses to end-stage kidney disease, the incidence of complications and death increases and is closely associated with high medical costs [2, 3]. The KDIGO guidelines state that regular follow-up and standard management can effectively improve patient health outcomes, slow the rate of disease progression, and reduce the incidence of complications [4].

For systematically and comprehensively manage patients with CKD, we established the CKD Management Center in 2013, started to build the database in 2015, and constructed the information system in 2018, which solved the problems of work efficiency and data collection in patient management. As the transformation of medical model to patient-centered management, ways to improve patients’ self-management ability has become a topical research issue. Smartphone apps have been shown to be used as an aid to facilitate the self-management of patients with chronic diseases such as COPD, asthma, heart disease and diabetes [5–9]. Previous studies have shown that smartphone app, as an auxiliary tool in patient remote follow-up management, can achieve the ideal effect of disease control, reduce complications, and improve treatment compliance and satisfaction in patients with CKD [10]. Therefore, based on the theory of patient self-management, we developed an APP called SuYi to facilitate long-term case management of patients with CKD in conjunction with traditional outpatient follow-up in 2019.

We designed a retrospective study to examine the impact of remote follow-up with a mobile application on patient outcomes, including laboratory data associated with renal function and incidence of renal endpoint events. Additionally, the study seeks to provide insights on the effectiveness of a hybrid approach to patient management that includes both traditional outpatient follow-up and remote follow-up with a mobile application. The results of this study can have significant implications for remote patient management and may inform the development of better strategies to manage CKD in a cost-effective and efficient manner.

## Methods

### Mobile app development

The agile model is a widely known software development methodology used as a guiding framework for building and testing application prototypes. The model consists of four stages (conception, initiation and analysis, design and construction, testing and deployment) that describe the overall process of software development.

#### Conception stage

Theoretical and empirical knowledge demonstrates that the self-management “process” is a major factor affecting adherence to chronic disease management, medical social resource utilization, and patient quality of life. Therefore, the core design, content and function of the Suyi App are key conceptual elements of self-management. Therefore, we formed a development team with doctors, nurses, engineers, and patients to create a list of tasks and procedures for the development of apps. The list was revised and grouped according to common CKD self-management goals in the clinical and research literature (Table 1), and operation systems such as IOS and Android wereselected for prototype development.

#### Initiation and analysis stage

ThinMed medical technology brings together a software development team (including engineers and designers) by four computer science practitioners, one algorithm engineer, one architect and one graphic designer developing the APP prototype. Continuously updated according to clinical needs and the next phase of application development and testing.

#### Design and construction stage

**Fig. 1:**
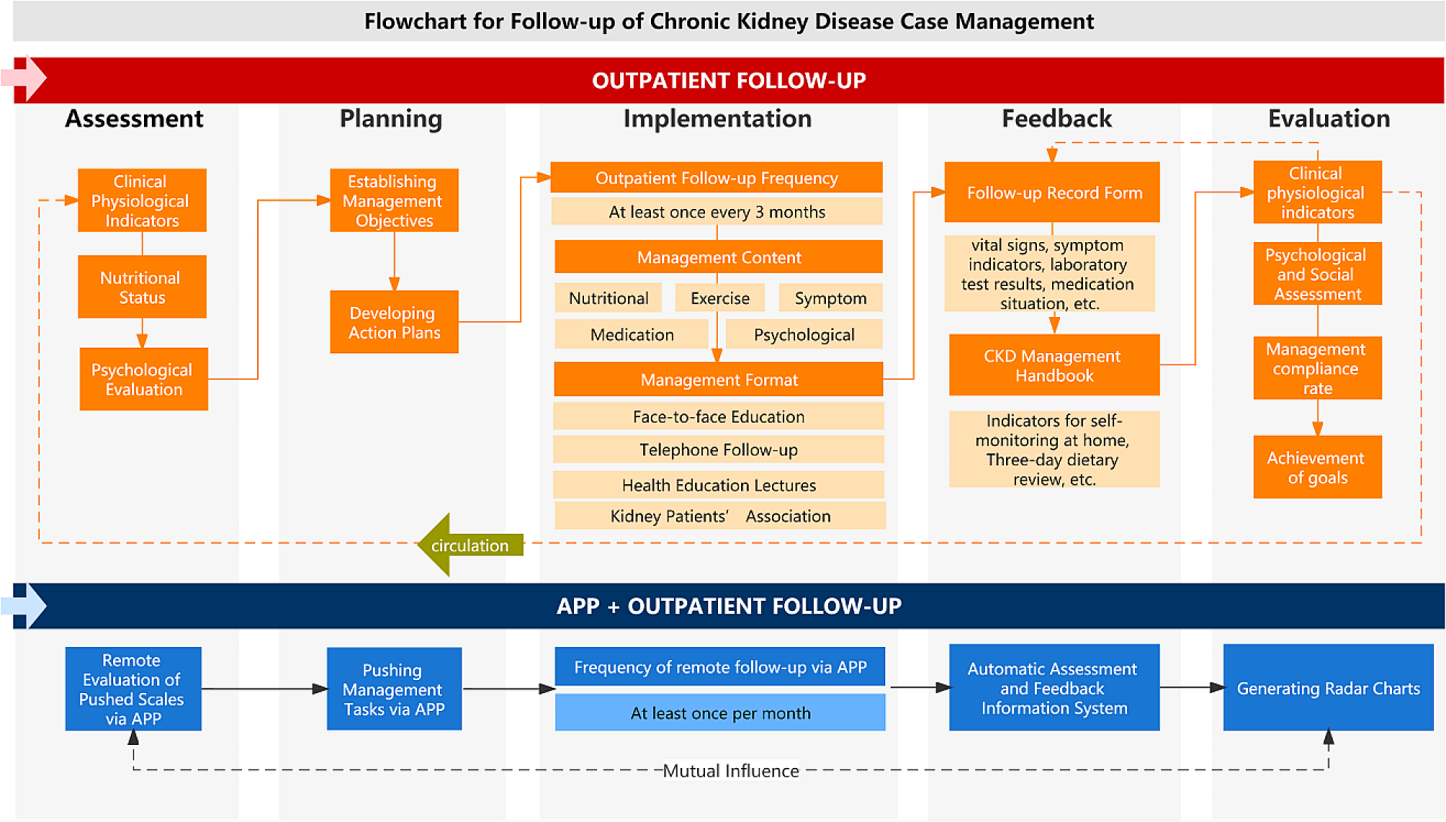
Flowchart of case management and follow-up for two patient groups

Based on the self-management process concept, the main objectives of the app design are (1) to simplify the patient‒physician communication process and (2) to assist patients in completing their home self-management more effectively. The design features of the app include the following: (1) scheduled follow-up appointments section; (2) task management push section; (3) practical knowledge push section; (4) automatic evaluation feedback section; (5) patient education live feature section; and (6) risk warning processing section. Figure 1 shows the screen diagram of the APP, and the outline of the process of the patient using the APP under the guidance of the health care provider.

#### Testing and deployment stage

A multidisciplinary CKD management team, development engineers and solution architects reviewed the app, asked questions, and then revised it again. Next, 10 patients (5 male and 5 female adults) were selected to complete the test using the app, following which they were asked for their opinions on improvements to the APP. Based on patient feedback, the font was enlarged, the sliding bar was changed to a numeric input keyboard, and occasional disconnections and flashbacks were resolved. Prior to the feasibility study, the APP was copyrighted (2020SR0835730). Additionally, approval from the institutional review board was obtained (2018-R006-01).

**Table 1:** Content of Suyi App

The Suyi APP Goals and Behaviors
1.The Suyi APP system contains multiple modules that cooperate with each other of:
• In hospital clinical terminal system
• App server
• Patient APP
2. Physical sign report and evaluation
• Blood pressure
• Blood sugar
• Heart rate and pulse
• Height and weight
3. Healthy learning and feedback
• Patient education course
• Health tasks
• Scale filling
• Questionnaire collection
4. Calculation and warning
• Alert parameter configuration
• Daily patient data back calculation
• Early warning feedback of clinical system
5. Algorithm and learning
• Photo taking and uploading of inspection list outside the hospital
• OCR image recognition
• Calculate and learn correction

### The use of Suyi APP

The Suyi APP encompasses core modules including personal information management, task assignment for disease management, reminders for follow-up appointments, physiological and psychological assessments, health education, outpatient medication management, home-based nutritional management, live patient education sessions, assessment feedback, and risk alert systems. The utilization process is as follows: Patients are enrolled in the CKD Management Center, where a medical record is established. The Suyi APP is installed on the patient’s mobile device, synchronizing in-hospital diagnostic and examination data to the APP interface. Nurses then dispatch home management task plans, patient education materials, assessment scales, and schedule live patient education sessions to the patients via the APP. Patients receive these management tasks and upload their home management data, such as body temperature, weight, blood pressure, and blood sugar levels, to the APP. They also access the educational materials and complete corresponding knowledge point assessments. Full-time nurses, on the PC end, receive the uploaded information from the patient’s APP, conduct a comprehensive evaluation, and provide feedback on the assessment.

### Study design

The study was a retrospective cohort study, the primary objective was to assess the effects of two follow-up methods (“ APP + outpatient follow-up “and"traditional outpatient follow-up “) improves laboratory data associated with renal function, reduces all-cause mortality, reduces the occurrence of renal replacement(hemodialysis(HD) /peritoneal dialysis(PD) / renal transplant(RT)) or avoids the initiation of urgent dialysis(defined as use of temporary catheters when initial hemodialysis).

### Setting

Patients enrolled in the CKD management center received long-term follow-up and management services from a dedicated nurse based on a case management model. The center was established in 2013, headed by one nurse director and one senior physician, and staffed with five full-time nurses. A kidney disease care clinic was in operation seven days a week (concurrently with a physician clinic), where all consultations were conducted by dedicated nurses. Case management included nutrition management, relevant diseases and complications management, medication management, symptom management, exercise management, and lifestyle management. The case management process consisted of assessment, planning, implementation, feedback, and evaluation. Patients were divided into two groups: the APP + outpatient follow-up group, where patients installed a mobile application and used it at least once a month, and had additional outpatient follow-ups at least once every three months; and the traditional outpatient follow-up group, where patients did not install the app and had outpatient follow-ups at least once every three months. All patients were followed up and managed according to the standard case management model (Fig. 1).

### Participants

**Fig. 2:**
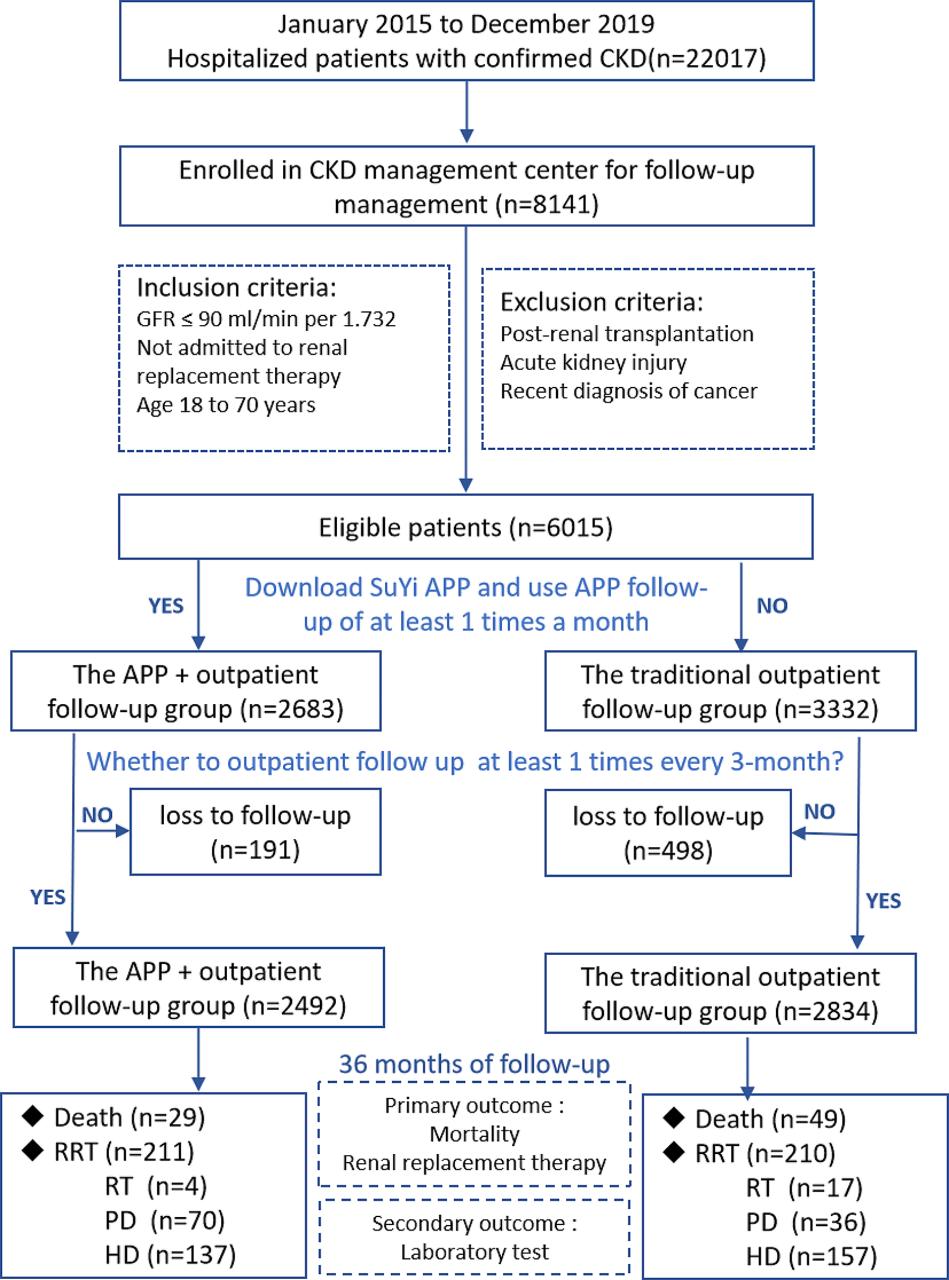
Flow chart of patient screening for inclusion in this study

Patients who were enrolled in the CKD management center for long-term follow-up from January 2015 to December 2019 without receiving renal replacement therapy and with CKD stages 1–5 were included in this study. The inclusion criteria were as follows: (1) diagnosis of CKD stage 1 to 5 [GFR < 90 ml/(min 1.73 m^2^)](the stage of CKD is based on the criteria proposed by the Kidney Disease: Improving Global Outcomes (KDIGO) organization), (2) age 18∼80 years old, and (3) no cognitive impairment, (4) with an outpatient follow-up at least once every 3 months. The exclusion criteria were as follows: (1) hemodialysis (HD), peritoneal dialysis (PD), or other treatment dialysis, renal transplantation (RT); (2) acute kidney injury; and (3) recent diagnosis of cancer,4) No outpatient follow-up was conducted for a period of more than 6 months. The collection of baseline data began in January 2020, and the study endpoint was December 2022. And refer to the flowchart below for specific screening process (Fig. 2).

### Outcome measurement

#### Laboratory data

Laboratory data associated with renal function include according to KDIGO: eGFR, Serum creatinine, Uric Acid, Calcium, Phosphorus, Kalium, Sodium (Na), Erythrocyte, Total Cholesterol, Triglyceride, Totol protein, Albumin, Parathormone, Hemoglobin.

#### All-cause mortality

Refers to the ratio of the number of deaths from all causes to the total number of patients in a certain period.

#### Incidence of entering renal replacement therapy

Refers to the ratio of the number of incidences of patients entering HD, PD, and RT to the total number of patients. The ratio of the number of incidences of patients entering HD, PD, and RT to those with CKD stage 4–5 at baseline was further analyzed.

#### The use rate of temporary dialysis catheter

The ratio between the number of patients using temporary catheters at the time of entry into HD and the total number of first-time HD patients. Reducing temporary dialysis catheter usage means avoiding the initiation of urgent dialysis.

### Bias

In the retrospective analysis, Patients who failed to return for follow-up at the center, lost contact, or withdrew from follow-up were considered lost to follow-up (no longer receiving CKD outpatient follow-ups at least once every three months).Baseline data analysis was performed using inverse probability treatment weighting to ensure comparability of baseline characteristics.

### Study size

Extracted through the in-hospital CKD management information system, eGFR < 90 ml/min/1.73 m^2^ was selected by engineers entering computer language, and a total of 6015 patients who met the inclusion exclusion criteria were extracted. 2683 people in the APP + outpatient group and 3332 people in the traditional outpatient group. The occurrence of renal endpoint events in both groups was collected by HIS system extraction with telephone follow-up, and laboratory test indexes were automatically extracted by the system.

### Statistical methods

The inverse probabilistic treatment weighting of the propensity score was used to balance the baseline health measures recorded in the comparison groups, including known indications for APP use [11, 12]. The propensity score was estimated by multivariable logistic regression with 164 covariates chosen a priori. Patients in the reference group were weighted. This method produces a weighted pseudo sample of patients in the reference group with the same distribution of measured covariates as the exposure group [13]. Standardized differences between unweighted and weighted samples were used to compare differences in baseline characteristics between groups (differences > 10% were considered meaningful). Weighted risk ratios and 95% CIs were obtained by modified Poisson regression, and weighted risk differences and 95% CIs were obtained by a binomial regression model with an identity link function. Two-tailed *P* values less than.05 were interpreted as statistically significant. Because multiple comparisons can lead to type I errors, the results of secondary, subgroup, and sensitivity analyses should be interpreted as exploratory analyses. Analyses were conducted using SAS statistical software, version 9.4 (SAS Institute Inc).

## Results

### Study participants

From January 2015 to December 2019, 6015 of 8141 patients in the CKD management center met the selection criteria for inclusion in the study, they were divided into two groups according to whether they downloaded and used the APP(used it at least once a month), of whom 3332 people were enrolled in the CKD management center to receive APP + outpatient follow-up and 2683 patients were managed by traditional outpatient follow-up. During the follow-up period from January 2020 to December 2022, 191 patients were lost to follow-up in the APP + outpatient follow-up group, including 107 patients who chose to return for local follow-up, 73 patients who did not return for follow-up, and 11 patients who were lost to contact (unable to be reached by phone). In the traditional outpatient follow-up group, 498 patients were lost to follow-up, including 203 patients who chose to return for local follow-up, 276 patients who did not return for follow-up, and 19 patients who were lost to contact. At the end of the 36-month follow-up period, 2942 patients in the APP + outpatient follow-up group and 2834 patients in the traditional outpatient follow-up group were included in the analysis (Fig. 2).

### Descriptive data

**Fig. 3:**
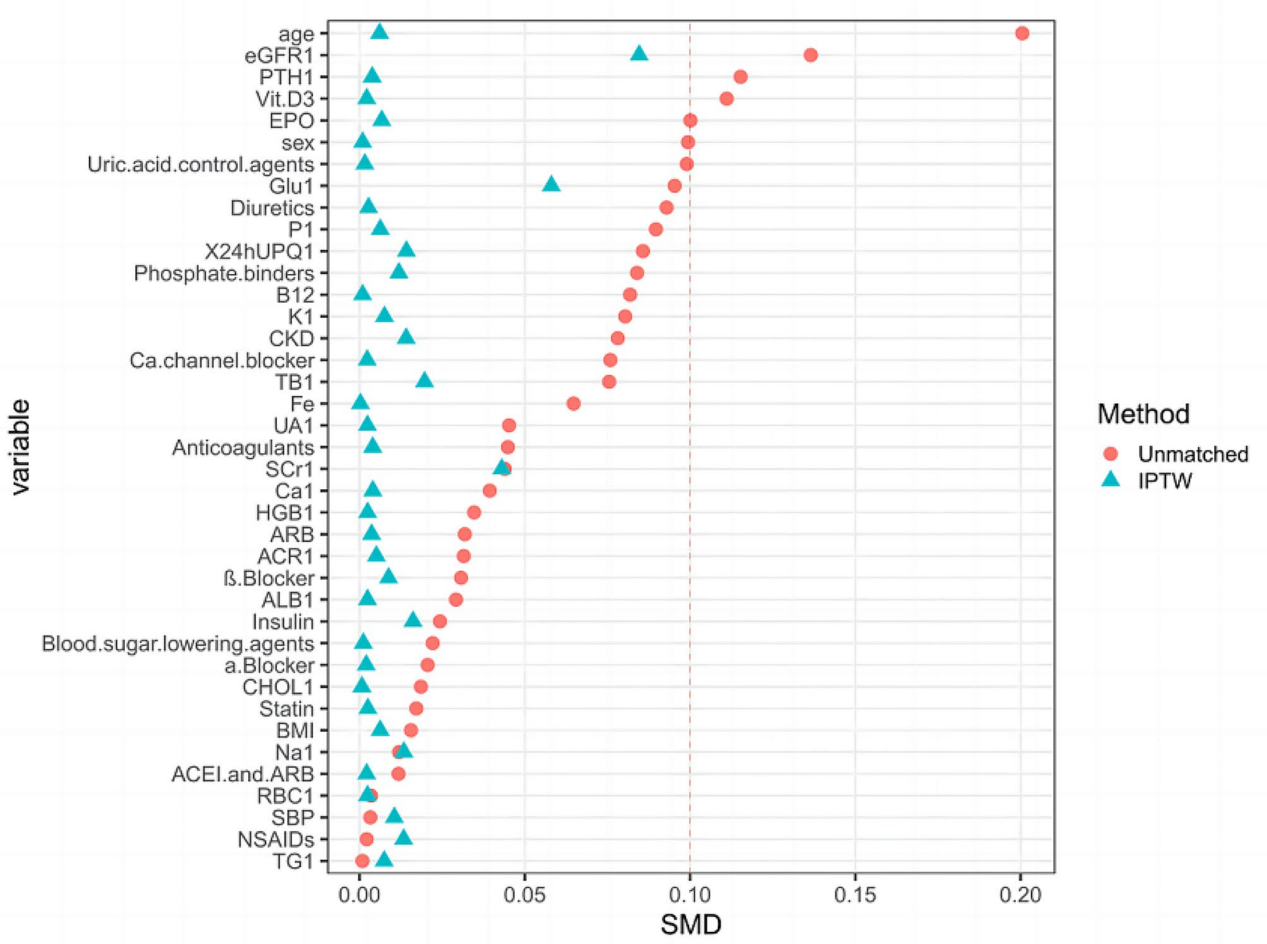
Standardized differences of each index before and after matching

The primary cohort consisted of 2492 patients (median, 45 years [IQR, 32–53]) in the APP + outpatient group and 2834 patients (median, 46 years [IQR, 34–56]) in the traditional outpatient group. After IPTW virtualization matching.(Fig. 3), the final matched APP + outpatient group consisted of 2489 cases (median, 46 years [IQR, 33–55]) and 2850 cases (median, 45 years [IQR, 33–55]) in the traditional outpatient group. Before weighting, all standardized differences were less than 10% except for age, EGFR1, PTH1, and Vit. D3, and after weighting, all standardized differences were less than 10% (Table 2).

**Table 2:** Baseline characteristics of the study with the patient

Variable	Observed cohort			IPTW cohort		
	APP+Outpatient *N*=2492	Traditional Outpatient *N*=2834	ASD	APP+Outpatient *N*=2489	Traditional Outpatient *N*=2850	ASD
Age, median(IQR), y	45.0 [32.0, 53.0]	46.0 [34.0, 56.0]	0.201	46.0 [33.0, 55.0]	45.0 [33.0, 55.0]	0.006
Male sex, n(%)	1394(55.9)	1445(51.0)	0.099	1328.1 (53.4)	1519.6(53.3)	0.001
BMI, median(IQR), kg/m^2^	23.1 [20.8, 25.6]	23.1 [20.9, 25.7]	0.016	23.1 [20.9, 25.5]	23.1 [20.8, 25.7]	0.006
CKD stages						
CKD 1	815(32.7)	1008(35.6)	0.078	855(34.3)	978(34.3)	0.001
CKD 2	85(23.5)	635(22.4)	0.029	577(23.2)	655(23.0)	0.003
CKD 3a	310(12.4)	370(13.0)	0.016	309(12.4)	355(12.5)	0.002
CKD 3b	283(11.4)	309(10.9)	0.015	281(11.3)	321(11.3)	0.001
CKD 4	302(12.1)	325(11.5)	0.022	291(11.7)	330(11.6)	0.002
CKD 5	197(7.9)	187(6.6)	0.032	176(7.1)	211(7.4)	0.005
**Laboratory data**,						
**median(IQR)**						
SBP	122[114, 136]	121 [115, 135]	0.003	122[114, 136]	121 [115, 135]	0.011
eGFR	65.0[33.0, 98.0]	73.0[39.3, 102]	0.137	67.0 [34.0, 98.0]	71.1 [37.0, 102]	0.085
Serum creatinine	107 [75.7, 181]	98.6 [70.4, 156]	0.044	103.3 [74.3, 172.6]	101.4 [71.5, 167.0]	0.043
Uric Acid	394.5[320, 470.4]	389.9 [316.1, 468.7]	0.045	390.7[316.2, 466.6]	391.8 [318.7, 471.0]	0.002
Calcium	2.29 [2.17, 2.38]	2.29 [2.18, 2.38]	0.039	2.29 [2.17, 2.38]	2.28 [2.18, 2.38]	0.004
Phosphorus	1.14 [0.99, 1.31]	1.12 [0.98, 1.27]	0.090	1.13 [0.98, 1.30]	1.12 [0.99, 1.29]	0.006
Kalium	4.11 [3.86, 4.46]	4.09 [3.81, 4.39]	0.080	4.09 [3.84, 4.43]	4.11 [3.82, 4.42]	0.008
Sodium (Na)	139.7 [138.1, 141.2]	139.8 [138.1, 141.2]	0.012	139.7[138.0, 141.3]	139.7[138.0, 141.2]	0.013
Erythrocyte	4.29 [3.79, 4.83]	4.30 [3.84, 4.78]	0.003	4.29 [3.81, 4.81]	4.29 [3.83, 4.79]	0.002
Total Cholesterol	4.64 [3.91, 5.56]	4.72 [4.00, 5.67]	0.019	4.69 [3.94, 5.59]	4.69 [3.96, 5.65]	0.001
Triglyceride	1.56 [1.11, 2.28]	1.51 [1.07, 2.26]	0.001	1.58 [1.11, 2.31]	1.51 [1.06, 2.25]	0.007
Totol protein	69.9[62.9, 75.0]	68.7[62.8, 73.8]	0.076	69.5 [62.4, 74.6]	69.3[63.1, 74.3]	0.02
Albumin	42.2[37.2, 45.6]	42.2 [37.6, 45.3]	0.029	42.2[37.1, 45.6]	42.1[37.3, 45.3]	0.002
Parathormone	62.5[41.4, 105.9]	56.5 [36.7, 93.5]	0.115	61.1 [40.5, 100.3]	58.0 [37.5, 98.1]	0.004
Hemoglobin	128.0 [111.0, 143.0]	129.0 [114.0, 142.0]	0.035	129.0 [113.0, 143.0]	128.0 [113.0, 142.0]	0.002
**Medication, n (%)**						
ACEI and ARB	107(4.3)	115(4.1)	0.012	105(4.2)	119(4.2)	0.002
ARB	1334(53.5)	1472(51.9)	0.032	1314(52.8)	1510(53.0)	0.004
Ca channel blocker	664(26.6)	852(30.1)	0.076	711(28.6)	818(28.7)	0.002
α-Blocker	6(0.2)	10(0.4)	0.021	8(0.3)	9(0.3)	0.002
β-Blocker	367(14.7)	387(13.7)	0.031	349(14.0)	408 (14.3)	0.009
NSAIDs	256(10.3)	293(10.3)	0.002	254(10.2)	303(10.6)	0.013
Vit D3	803(32.2)	1063(37.5)	0.111	877(35.3)	1002(35.1)	0.002
Statin	459(18.4)	541(19.1)	0.017	475(19.1)	541(19.0)	0.003
Phosphate binders	76(3.0)	50(1.8)	0.084	62(2.5)	77(2.7)	0.012
Diuretics	385(15.4)	347(12.2)	0.093	342(13.7)	389(13.7)	0.003
Uric acid control agents	1207(48.4)	1233(43.5)	0.099	1144(46.0)	1312(46.0)	0.002
EPO	370(14.8)	325(11.5)	0.100	322(12.9)	375(13.2)	0.007
Fe	95(3.8)	146(5.2)	0.065	112(4.5)	129(4.5)	<0.001
Blood sugar lowering agents	151(6.1)	187(6.6)	0.022	157(6.3)	180 (6.3)	0.001
Insulin	92(3.7)	92(3.2)	0.024	94(3.8)	99(3.5)	0.016
B12	71(2.8)	124(4.4)	0.082	92(3.7)	105(3.7)	0.001
Anticoagulants	1525(61.2)	1672(59.0)	0.045	1505(60.5)	1718(60.3)	0.004

Abbreviation: IPTW, inverse probability treatment weighting; ASD, absolute standardized difference; IQR, inter-quartile range; BMI, body mass index; SBP: systolic blood pressure; CKD, chronic kidney disease; ACEI, angiotensin converting enzyme inhibitor; ARB, angiotensin receptor blocker; ACR, Albumin-to-creatinine ratio; EPO, erythropoietin; NASIDs, non-steroidal anti-inflammatory drugs

### Evaluation outcomes

#### Laboratory data

The Wilcoxon signed-rank test indicates that the eGFR of patients in the APP + outpatient group increased after follow-up, while the eGFR of the traditional outpatient group decreased. Moreover, the Mann-Whitney test revealed differences in the increase of eGFR before and after follow-up between the two groups (*P* =.03). When compared to the traditional outpatient group, the APP + outpatient group showed significant improvements in laboratory data such as Phosphorus, Sodium, Triglyceride and Hemoglobin (*P* < .05) (Table 3).

**Table 3:** Comparison laboratory data before and after follow-up between the two groups

Laboratory data,	APP+Outpatient			Traditional Outpatient			*P* value^b^
Median(IQR)	Before	After	*P* value^a^	Before	After	*P* value^a^	
Mean arterial pressure	95.2[86.7-102.7]	-	-	93.3[86.7-101.4]	-	-	<0.001
eGFR	65.0[33.0-98.0]	66.0[34.0-98.0]	<0.001	73.0[39.3-102.0]	72.6[40.5-102.0]	0.001	0.03
Serum creatinine	106.9[75.7-181.4]	106.0[75.6-182.0]	<0.001	98.6[70.4-155.8]	100.0[70.8-161.9]	<0.001	0.3
Uric Acid	394.5[320.0-470.4]	383.2[309.6-464.0]	<0.001	389.9[316.1-468.7]	388.0[313.3-465.5]	0.42	<0.001
Calcium	2.3[2.2-2.4]	2.3[2.2-2.4]	0.08	2.3[2.2- 2.4]	2.3[2.2-2.4]	<0.001	0.97
Phosphorus	1.1[1.0-1.3]	1.1[1.0-1.3]	<0.001	1.1[1.0-1.3]	1.1[1.0-1.3]	<0.001	<0.001
Kalium	4.1[3.9-4.5]	4.1[3.9-4.5]	0.01	4.1[3.8-4.4]	4.1[3.8-4.4]	0.82	0.01
Sodium (Na)	139.7[138.1-141.2]	139.2[137.6-140.8]	<0.001	139.8[138.1-141.2]	139.5[137.9-141.0]	<0.001	<0.001
Erythrocyte	4.3 [3.8-4.8]	4.3[3.7-4.8]	0.04	4.3 [3.8-4.8]	4.3[3.8-4.8]	<0.001	0.11
Total Cholesterol	4.6[3.9-5.6]	4.6[3.9-5.5]	<0.001	4.7[4.0-5.7]	4.7[4.0-5.7]	0.35	0.12
Triglyceride	1.6[1.1-2.3]	1.5[1.1-2.2]	<0.001	1.5 [1.1-2.3]	1.5[1.1-2.3]	0.7	0.002
Totol protein	69.9[62.9- 75.0]	68.9[62.3-73.4]	<0.001	68.7[62.8-73.8]	67.8[61.8-72.6]	<0.001	0.01
Albumin	42.2[37.2-45.6]	42.1[37.4-45.3]	0.17	42.2[37.6-45.3]	42.1[37.7-45.2]	0.37	0.18
Parathormone	62.5[41.4-105.9]	65.6[42.5-115.9]	0.001	56.5[36.7-93.5]	58.0[37.2-97.1]	0.08	0.08
Hemoglobin	128.0[111.0- 143.0]	128.0[111.0-145.0]	0.06	129.0[114.0-142.0]	128.5[113.0-142.0]	0.09	0.01

aWilcoxon signed-rank test; bMann-Whitney test.

### All-cause mortality outcomes

Before matching, there were 29 deaths (1.2%) in the APP + outpatient group and 49 deaths (1.7%) in the traditional outpatient group, with a rate difference of -0.57 (95% CI: -1.2 to 0.07) between the two groups, which was not significantly different (*P* = .09) (Table 4). After matching, there were 34 deaths (1.4%) in the APP + outpatient group and 46 deaths (1.6%) in the traditional outpatient group, with a rate difference of -0.26 (95% CI: -0.91 to 0.39), which was not significantly different (*P* = .49) (Table 4).

**Table 4:** Comparison endpoint outcomes after follow-up between the two groups

Outcome	Observed cohort					IPTW cohort				
	No. of Events(%)		RD(95%CI)	HR(95%CI)	*P* value	No. of Events(%)		RD(95%CI)	HR(95%CI)	*P* value
	APP+Outpatient *N*=2492	Traditional Outpatient *N*=2834				APP+Outpatient *N*=2489	Traditional Outpatient *N*=2850			
Mortality	29(1.2)	49(1.7)	-0.57(-1.20, 0.07)	0.67(0.43,1.06)	0.09	34(1.4)	46(1.6)	-0.26(-0.91, 0.39)	0.84(0.52, 1.36)	0.49
RRT	211(8.5)	210(7.4)	1.06(-0.40, 2.51)	1.15(0.95,1.39)	0.15	190(7.6)	245(8.6)	-0.99(-2.45, 0.48)	0.88(0.72, 1.08)	0.23
HD	137(5.5)	157(5.5)	-0.04(-1.27, 1.19)	1.00(0.79,1.25)	0.97	127(5.1)	171(6.0)	-0.90(-2.13, 0.32)	0.85(0.66, 1.07)	0.17
PD	70(2.8)	36(1.3)	1.54(0.77, 2.31)	2.23(1.49,3.34)	<0.001	58(2.3)	56(1.9)	0.40(-0.38, 1.18)	1.21(0.77 ,1.89)	0.41
RT	4(0.2)	17(0.6)	-0.44(-0.76,-0.11)	0.27(0.09,0.80)	0.02	4(0.2)	19(0.7)	-0.49(-0.83, -0.15)	0.26(0.09, 0.81)	0.02

Abbreviation: IPTW, inverse probability treatment weighting; RD, risk difference; HR, hazard ratio; RRT, renal replacement therapy; HD, hemodialysis; PD, peritoneal dialysis; RT, renal transplantation

### Renal replacement therapy outcomes

Renal replacement therapy consisted of hemodialysis, peritoneal dialysis, and renal transplantation. Before matching, overall replacement therapy had 211 cases (8.5%) in the APP + outpatient group and 210 cases (7.4%) in the traditional outpatient group, *P* = .15, which was not significant (Table 4). After matching, overall replacement therapy was available in 190 cases (1.4%) in the APP + outpatient group and 245 cases (8.6%) in the traditional outpatient group (*P* = .23), which was not significant (Table 4).

### Temporary dialysis catheter outcomes

**Fig. 4:**
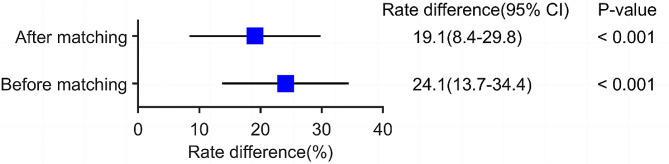
Differences in the temporary dialysis catheter between APP and control groups before and after matching

Before matching, a temporary dialysis catheter was performed in 60 cases (43.8%, [60/137]) in the APP + outpatient group and 31 cases (19.7%, [31/157]) in the traditional outpatient group, with a rate difference and 95% confidence interval of 24.1% [13.7-34.4%], *P* < .001, and the rate of tube placement was significantly higher in the APP + outpatient group than in the traditional outpatient group (Table 5). After matching, a temporary dialysis catheter was placed in 54 patients in the APP + outpatient group (42.5%, [54/127]) and 40 patients in the traditional outpatient group (23.4%, [40/171]), with a rate difference and 95% confidence interval of 19.1% [8.4-29.8%], *P* < .001, and the rate of tube placement in the APP + outpatient group was still significantly higher than that in the traditional outpatient group (Fig. 4).

**Table 5:** Comparison of temporary catheter use rate after follow-up between the two groups in CKD 4-5

Group	Mortality	RRT				Group	Access selection during initial HD	
		Total	HD	PD	RT		CVC	TCC or AVF
APP+Outpatient *N*=512	20(3.9)	150(29.2)	114(76.0)	25(16.6)	11(7.3)	APP+Outpatient *N*=114	60(52.6)	54(47.3)
Traditional Outpatient *N*=499	13(2.6)	153(30.6)	101(66.0)	49(32.0)	3(1.9)	Traditional Outpatient *N*=101	31(30.6)	70(69.3)
Pearson’s chi-squared test	1.36	0.11	13.11			Pearson’s chi-squared test	10.59	
*P* value	0.24	0.73	<0.001			*P* value	<0.001	

Abbreviation: RRT, renal replacement therapy; HD, hemodialysis; PD, peritoneal dialysis; RT, renal transplantation; CVC, central venous catheter; TCC, tunnel-cuffed catheter; AVF, Arteriovenous Fistula

## Discussion

This study aims to assess how the integration of the APP with outpatient follow-up affects clinical indicators and outcomes in individuals with CKD. As far as we know, this is the largest clinical trial evaluating remote management in patients with CKD. Based on the findings, there were no significant discrepancies noted in the rates of mortality or primary outcome of renal replacement therapy between the group that received APP + outpatient intervention and the traditional outpatient group. In contrast, the APP + outpatient group exhibited noteworthy enhancements in laboratory data such as Phosphorus, Sodium, Triglyceride and Hemoglobin. However, it is important to highlight that patients in advanced stages of CKD (stages 4–5) require strong emphasis on traditional outpatient follow-ups to adequately prepare for renal replacement therapy and reduce the need for temporary catheter usage when opting for HD.

### The necessity of long-term follow-up management for CKD patients

CKD is a significant public health concern and poses a burden on healthcare systems due to its high incidence and prevalence, high rates of disability, substantial medical costs, and low levels of awareness. The Kidney Disease Improving Global Outcomes (KIDIGO) guidelines suggest that regular follow-up and comprehensive management can slow the progression of CKD alongside drug therapy [14]. Improving lifestyle behaviors, such as controlling salt or protein intake, adhering to medications and regular physical exercise, have been known to reduce the risk and progression [15, 16]. In 2013, our project team established a CKD management center with a follow-up system and explored various management models [16]. Five full-time nurses provided long-term outpatient follow-up and management services to enrolled patients, successfully achieving the goal of delaying CKD progression and improving patients’ quality of life. However, the popularity of the Internet posed a challenge to the original CKD outpatient follow-up model. It was imperative to explore and leverage “Internet + " technology for remote follow-up [17]. Therefore, we developed the Suyi APP based on patients’ self-management capabilities, which enabled remote management during the epidemic control measures. However, due to the popularity of the Internet, it poses challenges to the traditional outpatient model.

### Utilizing APP for outpatient follow-up can help improve patient health outcomes

APP interventions have the benefits of being multifunctional, including communicating with and collecting information from users as well as providing interactive experiences [18, 19]. APP interventions provide a platform for the delivery of adherence interventions that are considered to be highly customizable to each unique individual, available at low cost, and easily accessible [20]. Using internet technology to develop information means for multichannel follow-up intervention is of great significance to prevent the occurrence of CKD and the progression of end-stage kidney disease [21]. Mohammed’s study presents the Adaptive Federated Reinforcement Learning-Enabled System (AFRLS) for Internet of Things(IoT) consumers’ kidney disease image processing, reduces the time tasks need to be delayed [22]. Tuot believes that telemedicine could improve knowledge and behavior changes for CKD patients and primary care providers [23]. Esmaeil’ study shows that a mobile-based self-management system can lead to adherence to the medication regimens and promotion of the health of people living with HIV (PLWH) [24, 25]. Kelly’s study on coaching to promote healthy eating in patients by phone and text message was an available intervention that appears feasible for supporting dietary self-management in stage 3–4 CKD [26]. Blood pressure [27] and diet quality [28] of early patients were effectively improved after short-term, intensive and remote dietary APP intervention, decreased acid production and potentially mitigated CKD progression [29]. These findings demonstrate that app intervention can improve patients’ self-care and medication adherence by providing appropriate lifestyle, nutrition and medication guidance. The Suyi APP for CKD, which is based on the patient’s self-management capabilities, includes modules for health data tracking, receiving medication information, appointment reminders, and home self-management, enabling patients to effectively self-manage at home. By combining internet technology with traditional outpatient follow-up management, coordination between healthcare providers and patients is improved, providing patients with more comprehensive healthcare services and significantly improving their clinical outcomes. However, in the retrospective selection of patients for the program, we found that its success depends on several key factors, including the design and functionality of the APP, patient health education, and the level of support and follow-up provided by healthcare providers. Additionally, patient willingness to accept and use the APP is crucial. In conclusion, the development and application of the APP, along with outpatient follow-up management, can positively impact the health outcomes of CKD patients and improve nursing quality.

### CKD stages 4–5 require more frequent face-to-face outpatient follow-up care

In stage 4–5 of CKD, the kidneys are more severely damaged, often accompanied by uremia and other serious complications, including cardiovascular disease, osteoporosis, and anemia. When it is necessary to enter kidney replacement therapy, patients need to choose their treatment plan.The choice of RRT is a critical decision that has a great impact on the lives of both patients and their families, so it is advisable to practice shared decision-making when trying to choose the best treatment [30, 31]. Face-to-face health education for outpatient follow-up could facilitate more communication and build better relationships between patients, family members, and healthcare team members [32, 33]. For patients with ESKD entering HD, the best vascular access is an autologous arteriovenous endovascular fistula, a prosthetic vessel, or a temporary catheter if long-term vascular access is not ready at the time of entry into HD. Numerous studies have shown that the risk of bloodstream infection with a temporary catheter is 7.64 times higher than that with an autologous arteriovenous fistula [32], and the high rates of mortality immediately after ESKD were mostly accounted for by the HD with catheter population [33, 34]. Studies have demonstrated that participation in predialysis face to face education resulted in lower initiation of emergency dialysis through the use of temporary catheters [35]. The findings of this study confirm that the use of temporary catheters during the first hemodialysis is decreased in patients who have received traditional outpatient follow-up. Next, it is necessary to further develop an information-based decision-making support tool for renal replacement therapy.

### Limitations

The current study has several limitations. First, it was a single-center observational study, and the long-term efficacy of app intervention through our CKD management program could not be definitively demonstrated. Second, patients have different cultural literacy and abilities to acquire knowledge from apps, and many elderly patients cannot proficiently use apps. More data from patients are needed, and the long-term effects of app intervention need to be investigated in a larger cohort of patients. Additionally, future directions for telemedicine use and research and quality measurement need to be explored.

### Comparison with prior work

On the one hand, previous studies focused more on the development and verification of the effectiveness of APP in clinical use, without analyzing and comparing how to use APP in specific clinical situations. By comparing the use of the same APP in different clinical situations, this study confirmed that the APP should cooperate with clinical application. If APP is to be used for simple remote follow-up, its personalized functions and settings need to be further improved. On the other hand, most prior studies have relied on limited sample sizes, short follow-up times and different research objectives. To the best of our knowledge, our study is the largest clinical trial evaluating remote management in patients with CKD, and the follow-up time was up to 3 years.

## Conclusions

The development and application of an app combined with outpatient follow-up management can improve patient health outcomes. However, to ensure optimal preparation for kidney replacement therapy, patients in CKD stages 4–5 may require more frequent traditional outpatient follow-ups, and further develop an information-based decision-making support tool for renal replacement therapy.

This research provides evidence that the convergence of mobile health applications with conventional care can substantially improve health outcome. By integrating mobile applications into CKD management, we have identified several tangible benefits. These include heightened patient engagement through customized health monitoring and educational resources, the facilitation of prompt medical interventions through the real-time tracking of physiological indicators and symptoms, and the fostering of a more collaborative approach to chronic disease management. The application serves as a vital communication platform, bridging the gap between patients and healthcare providers.

Building on our findings, we propose a framework for the development of a sophisticated digital decision-making support tool for renal replacement therapy. This tool is designed to cater to the unique needs of CKD stages 4–5 patients. Looking ahead, our future research agenda includes conducting longitudinal studies to evaluate the enduring effects of app-based management on patient outcomes and overall quality of life. We also advocate for further exploration into the integration of cutting-edge analytics and artificial intelligence within mobile applications, with the goal of refining personalized patient care.

### Electronic supplementary material

Below is the link to the electronic supplementary material.

Supplementary Material 1

Supplementary Material 2

Supplementary Material 3

Supplementary Material 4

Supplementary Material 5

## Data Availability

The datasets used and/or analysed during the current study are available from the corresponding author on reasonable request.

## Abbreviations

CKD: Chronic Kidney Disease
APP: Application program
HD: Hemodialysis
PD: Peritoneal Dialysis
RT: Renal Transplant
IPTW: Inverse Probability of Treatment Weighting
ASD: absolute standardized difference
IQR: Inter-quartile range
BMI: Body mass index
SBP: Systolic blood pressure
ACEI: Angiotensin converting enzyme inhibitor
ARB: Angiotensin receptor blocker
ACR: Albumin-to-creatinine ratio
EPO: Erythropoietin
NASIDs: Non-steroidal anti-inflammatory drugs
RD: Risk difference
HR: Hazard ratio
RRT: Renal replacement therapy
CVC: Central venous catheter
TCC: Tunnel-cuffed catheter
AVF: Arteriovenous Fistula
