# Supplementary material for: Impact of mobile application and outpatient follow-up on renal endpoints and physiological indices in patients with chronic kidney disease: a retrospective cohort study in Southwest China
Source: BMC Med Inform Decis Mak. 2024 Jun 12;24:163. doi: 10.1186/s12911-024-02567-3 (PMC11167892; doi:10.1186/s12911-024-02567-3)
Supplement: Supplementary file 1 — Supplementary Material 1 [file 12911_2024_2567_MOESM1_ESM.docx]

**Table 3. Comparison laboratory data before and after follow-up between the two groups**

| Laboratory data,  Median(IQR) | **APP+Outpatient** | | | **Traditional** **Outpatient** | | | ***P* value^b^** |
| --- | --- | --- | --- | --- | --- | --- | --- |
|  | **Before** | **After** | ***P* value^a^** | **Before** | **After** | ***P* value^a^** |  |
| Mean arterial pressure | 95.2[86.7-102.7] | - |  | 93.3[86.7-101.4] | - |  | <.001 |
| eGFR | 65.0[33.0-98.0] | 66.0[34.0-98.0] | <.001 | 73.0[39.3-102.0] | 72.6[40.5-102.0] | .001 | .03 |
| Serum creatinine | 106.9[75.7-181.4] | 106.0[75.6-182.0] | <.001 | 98.6[70.4-155.8] | 100.0[70.8-161.9] | <.001 | .30 |
| Uric Acid | 394.5[320.0-470.4] | 383.2[309.6-464.0] | <.001 | 389.9[316.1-468.7] | 388.0[313.3-465.5] | .42 | <.001 |
| Calcium | 2.3[2.2-2.4] | 2.3[2.2-2.4] | .08 | 2.3[2.2- 2.4] | 2.3[2.2-2.4] | <.001 | .97 |
| Phosphorus | 1.1[1.0-1.3] | 1.1[1.0-1.3] | <.001 | 1.1[1.0-1.3] | 1.1[1.0-1.3] | <.001 | <.001 |
| Kalium | 4.1[3.9-4.5] | 4.1[3.9-4.5] | .01 | 4.1[3.8-4.4] | 4.1[3.8-4.4] | .82 | .01 |
| Sodium (Na) | 139.7[138.1-141.2] | 139.2[137.6-140.8] | <.001 | 139.8[138.1-141.2] | 139.5[137.9-141.0] | <.001 | <.001 |
| Erythrocyte | 4.3 [3.8-4.8] | 4.3[3.7-4.8] | .04 | 4.3 [3.8-4.8] | 4.3[3.8-4.8] | <.001 | .11 |
| Total Cholesterol | 4.6[3.9-5.6] | 4.6[3.9-5.5] | <.001 | 4.7[4.0-5.7] | 4.7[4.0-5.7] | .35 | .12 |
| Triglyceride | 1.6[1.1-2.3] | 1.5[1.1-2.2] | <.001 | 1.5 [1.1-2.3] | 1.5[1.1-2.3] | .70 | .002 |
| Totol protein | 69.9[62.9- 75.0] | 68.9[62.3-73.4] | <.001 | 68.7[62.8-73.8] | 67.8[61.8-72.6] | <.001 | .01 |
| Albumin | 42.2[37.2-45.6] | 42.1[37.4-45.3] | .17 | 42.2[37.6-45.3] | 42.1[37.7-45.2] | .37 | .18 |
| Parathormone | 62.5[41.4-105.9] | 65.6[42.5-115.9] | .001 | 56.5[36.7-93.5] | 58.0[37.2-97.1] | .08 | .08 |
| Hemoglobin | 128.0[111.0- 143.0] | 128.0[111.0-145.0] | .06 | 129.0[114.0-142.0] | 128.5[113.0-142.0] | .09 | .01 |

^a^Wilcoxon signed-rank test; ^b^Mann-Whitney test.
