# Supplementary material for: Impact of mobile application and outpatient follow-up on renal endpoints and physiological indices in patients with chronic kidney disease: a retrospective cohort study in Southwest China
Source: BMC Med Inform Decis Mak. 2024 Jun 12;24:163. doi: 10.1186/s12911-024-02567-3 (PMC11167892; doi:10.1186/s12911-024-02567-3)
Supplement: Supplementary file 2 — Supplementary Material 2 [file 12911_2024_2567_MOESM2_ESM.docx]

**Table 1 Content of Suyi App**

The Suyi APP Goals and Behaviors

The Suyi APP system contains multiple modules that cooperate with each other of:

• In hospital clinical terminal system

• App server

• Patient APP

Physical sign report and evaluation

• Blood pressure

• Blood sugar

• Heart rate and pulse

• Height and weight

Healthy learning and feedback

• Propaganda course

• Health tasks

• Scale filling

• Questionnaire collection

Calculation and warning

• Alert parameter configuration

• Daily patient data back calculation

• Early warning feedback of clinical system

Algorithm and learning

• Photo taking and uploading of inspection list outside the hospital

• OCR image recognition

• Calculate and learn correction

**Table 1 Content of Suyi App**

| The Suyi APP Goals and Behaviors |
| --- |
| The Suyi APP system contains multiple modules that cooperate with each other of |
| • In hospital clinical terminal system  • App server  • Patient APP |
| Physical sign report and evaluation  • Blood pressure  • Blood sugar  • Heart rate and pulse  • Height and weight  Healthy learning and feedback  • Propaganda course  • Health tasks  • Scale filling  • Questionnaire collection  Calculation and warning  • Alert parameter configuration  • Daily patient data back calculation  • Early warning feedback of clinical system  Algorithm and learning  • Photo taking and uploading of inspection list outside the hospital  • OCR image recognition  • Calculate and learn correction |
