# Supplementary material for: Impact of mobile application and outpatient follow-up on renal endpoints and physiological indices in patients with chronic kidney disease: a retrospective cohort study in Southwest China
Source: BMC Med Inform Decis Mak. 2024 Jun 12;24:163. doi: 10.1186/s12911-024-02567-3 (PMC11167892; doi:10.1186/s12911-024-02567-3)
Supplement: Supplementary file 3 — Supplementary Material 3 [file 12911_2024_2567_MOESM3_ESM.docx]

**Table 2. Baseline characteristics of the study with the patient who use APP participated (N=2492) and did not participate (N=2834) in the outpatient follow-up**

| Variable | **Observed cohort** | | | **IPTW cohort** | | |
| --- | --- | --- | --- | --- | --- | --- |
|  | **APP+Outpatient**  N=2492 | **Traditional Outpatient**  N=2834 | **ASD** | **APP+Outpatient**  N=2489 | **Traditional** **Outpatient**  N=2850 | **ASD** |
| Age, median(IQR),y | 45.0 [32.0, 53.0] | 46.0 [34.0, 56.0] | 0.201 | 46.0 [33.0, 55.0] | 45.0 [33.0, 55.0] | 0.006 |
| Male sex, n(%) | 1394(55.9) | 1445(51.0) | 0.099 | 1328.1 (53.4) | 1519.6(53.3) | 0.001 |
| BMI,median(IQR), kg/m^2^ | 23.1 [20.8, 25.6] | 23.1 [20.9, 25.7] | 0.016 | 23.1 [20.9, 25.5] | 23.1 [20.8, 25.7] | 0.006 |
| CKD stages |  |  |  |  |  |  |
| CKD 1 | 815(32.7) | 1008(35.6) | 0.078 | 855(34.3) | 978(34.3) | 0.001 |
| CKD 2 | 585(23.5) | 635(22.4) | 0.029 | 577(23.2) | 655(23.0) | 0.003 |
| CKD 3a | 310(12.4) | 370(13.0) | 0.016 | 309(12.4) | 355(12.5) | 0.002 |
| CKD 3b | 283(11.4) | 309(10.9) | 0.015 | 281(11.3) | 321(11.3) | 0.001 |
| CKD 4 | 302(12.1) | 325(11.5) | 0.022 | 291(11.7) | 330(11.6) | 0.002 |
| CKD 5 | 197(7.9) | 187(6.6) | 0.032 | 176(7.1) | 211(7.4) | 0.005 |
| **Laboratory data,**  **median(IQR)** |  |  |  |  |  |  |
| SBP | 122[114, 136] | 121 [115, 135] | 0.003 | 122[114, 136] | 121 [115, 135] | 0.011 |
| eGFR | 65.0[33.0, 98.0] | 73.0[39.3, 102] | 0.137 | 67.0 [34.0, 98.0] | 71.1 [37.0, 102] | 0.085 |
| Serum creatinine | 107 [75.7, 181] | 98.6 [70.4, 156] | 0.044 | 103.3 [74.3, 172.6] | 101.4 [71.5, 167.0] | 0.043 |
| Uric Acid | 394.5[320., 470.4] | 389.9 [316.1, 468.7] | 0.045 | 390.7 [316.2, 466.6] | 391.8 [318.7, 471.0] | 0.002 |
| Calcium | 2.29 [2.17, 2.38] | 2.29 [2.18, 2.38] | 0.039 | 2.29 [2.17, 2.38] | 2.28 [2.18, 2.38] | 0.004 |
| Phosphorus | 1.14 [0.99, 1.31] | 1.12 [0.98, 1.27] | 0.09 | 1.13 [0.98, 1.30] | 1.12 [0.99, 1.29] | 0.006 |
| Kalium | 4.11 [3.86, 4.46] | 4.09 [3.81, 4.39] | 0.08 | 4.09 [3.84, 4.43] | 4.11 [3.82, 4.42] | 0.008 |
| Sodium (Na) | 139.7 [138.1, 141.2] | 139.8 [138.1, 141.2] | 0.012 | 139.7[138.0, 141.3] | 139.7[138.0, 141.2] | 0.013 |
| Erythrocyte | 4.29 [3.79, 4.83] | 4.30 [3.84, 4.78] | 0.003 | 4.29 [3.81, 4.81] | 4.29 [3.83, 4.79] | 0.002 |
| Total Cholesterol | 4.64 [3.91, 5.56] | 4.72 [4.00, 5.67] | 0.019 | 4.69 [3.94, 5.59] | 4.69 [3.96, 5.65] | 0.001 |
| Triglyceride | 1.56 [1.11, 2.28] | 1.51 [1.07, 2.26] | 0.001 | 1.58 [1.11, 2.31] | 1.51 [1.06, 2.25] | 0.007 |
| Totol protein | 69.9[62.9, 75.0] | 68.7[62.8, 73.8] | 0.076 | 69.5 [62.4, 74.6] | 69.3[63.1, 74.3] | 0.020 |
| Albumin | 42.2[37.2, 45.6] | 42.2 [37.6, 45.3] | 0.029 | 42.2[37.1, 45.6] | 42.1[37.3, 45.3] | 0.002 |
| Parathormone | 62.5[41.4, 105.9] | 56.5 [36.7, 93.5] | 0.115 | 61.1 [40.5, 100.3] | 58.0 [37.5, 98.1] | 0.004 |
| Hemoglobin | 128.0 [111.0, 143.0] | 129.0 [114.0, 142.0] | 0.035 | 129.0 [113.0, 143.0] | 128.0 [113.0, 142.0] | 0.002 |
| **Medication, n (%)** |  |  |  |  |  |  |
| ACEI and ARB | 107(4.3) | 115(4.1) | 0.012 | 105(4.2) | 119(4.2) | 0.002 |
| ARB | 1334(53.5) | 1472(51.9) | 0.032 | 1314(52.8) | 1510(53.0) | 0.004 |
| Ca channel blocker | 664(26.6) | 852(30.1) | 0.076 | 711(28.6) | 818(28.7) | 0.002 |
| α-Blocker | 6(0.2) | 10(0.4) | 0.021 | 8(0.3) | 9(0.3) | 0.002 |
| β-Blocker | 367(14.7) | 387(13.7) | 0.031 | 349(14.0) | 408 (14.3) | 0.009 |
| NSAIDs | 256(10.3) | 293(10.3) | 0.002 | 254(10.2) | 303(10.6) | 0.013 |
| Vit D3 | 803(32.2) | 1063(37.5) | 0.111 | 877(35.3) | 1002(35.1) | 0.002 |
| Statin | 459(18.4) | 541(19.1) | 0.017 | 475(19.1) | 541(19.0) | 0.003 |
| Phosphate binders | 76(3.0) | 50(1.8) | 0.084 | 62(2.5) | 77(2.7) | 0.012 |
| Diuretics | 385(15.4) | 347(12.2) | 0.093 | 342(13.7) | 389(13.7) | 0.003 |
| Uric acid control agents | 1207(48.4) | 1233(43.5) | 0.099 | 1144(46.0) | 1312(46.0) | 0.002 |
| EPO | 370(14.8) | 325(11.5) | 0.1 | 322(12.9) | 375(13.2) | 0.007 |
| Fe | 95(3.8) | 146(5.2) | 0.065 | 112(4.5) | 129(4.5) | <0.001 |
| Blood sugar lowering agents | 151(6.1) | 187(6.6) | 0.022 | 157(6.3) | 180 (6.3) | 0.001 |
| Insulin | 92(3.7) | 92(3.2) | 0.024 | 94(3.8) | 99(3.5) | 0.016 |
| B12 | 71(2.8) | 124(4.4) | 0.082 | 92(3.7) | 105(3.7) | 0.001 |
| Anticoagulants | 1525(61.2) | 1672(59.0) | 0.045 | 1505(60.5) | 1718(60.3) | 0.004 |

Abbreviation: IPTW, inverse probability treatment weighting; ASD, absolute standardized difference; IQR, inter-quartile range; BMI, body mass index; SBP: systolic blood pressure; CKD, chronic kidney disease; ACEI, angiotensin converting enzyme inhibitor; ARB, angiotensin receptor blocker; ACR, Albumin-to-creatinine ratio; EPO, erythropoietin; NASIDs, non-steroidal anti-inflammatory drugs
