# Supplementary material for: Impact of mobile application and outpatient follow-up on renal endpoints and physiological indices in patients with chronic kidney disease: a retrospective cohort study in Southwest China
Source: BMC Med Inform Decis Mak. 2024 Jun 12;24:163. doi: 10.1186/s12911-024-02567-3 (PMC11167892; doi:10.1186/s12911-024-02567-3)
Supplement: Supplementary file 4 — Supplementary Material 4 [file 12911_2024_2567_MOESM4_ESM.docx]

**Table 4. Comparison endpoint outcomes after follow-up between the two groups**

| **Outcome** | **Observed cohort** | | | | | **IPTW cohort** | | | | |
| --- | --- | --- | --- | --- | --- | --- | --- | --- | --- | --- |
|  | No. of Events(%) | | RD(95%CI) | HR(95%CI) | *P* value | No. of Events(%) | | RD(95%CI) | HR(95%CI) | *P* value |
|  | **APP+**  **Outpatient**  N=2492 | **Traditional** **Outpatient**  N=2834 |  |  |  | **APP+**  **Outpatient**  N=2489 | **Traditional** **Outpatient**  N=2850 |  |  |  |
| **Mortality** | 29(1.2) | 49(1.7) | -0.57(-1.20, 0.07) | 0.67(0.43,1.06) | .09 | 34(1.4) | 46(1.6) | -0.26(-0.91, 0.39) | 0.84(0.52, 1.36) | .49 |
| **RRT** | 211(8.5) | 210(7.4) | 1.06(-0.40, 2.51) | 1.15(0.95,1.39) | .15 | 190(7.6) | 245(8.6) | -0.99(-2.45, 0.48) | 0.88(0.72, 1.08) | .23 |
| HD | 137(5.5) | 157(5.5) | -0.04(-1.27, 1.19) | 1.00(0.79,1.25) | .97 | 127(5.1) | 171(6.0) | -0.90(-2.13, 0.32) | 0.85(0.66, 1.07) | .17 |
| PD | 70(2.8) | 36(1.3) | 1.54(0.77, 2.31) | 2.23(1.49,3.34) | <.001 | 58(2.3) | 56(1.9) | 0.40(-0.38, 1.18) | 1.21(0.77 ,1.89) | .41 |
| RT | 4(0.2) | 17(0.6) | -0.44(-0.76,-0.11) | 0.27(0.09,0.80) | .02 | 4(0.2) | 19(0.7) | -0.49(-0.83, -0.15) | 0.26(0.09, 0.81) | .02 |

Abbreviation: IPTW, inverse probability treatment weighting; RD, risk difference; HR, hazard ratio; RRT, renal replacement therapy; HD, hemodialysis; PD, peritoneal dialysis; RT, renal transplantation.
