# Supplementary material for: Impact of mobile application and outpatient follow-up on renal endpoints and physiological indices in patients with chronic kidney disease: a retrospective cohort study in Southwest China
Source: BMC Med Inform Decis Mak. 2024 Jun 12;24:163. doi: 10.1186/s12911-024-02567-3 (PMC11167892; doi:10.1186/s12911-024-02567-3)
Supplement: Supplementary file 5 — Supplementary Material 5 [file 12911_2024_2567_MOESM5_ESM.docx]

**Table 5. Comparison of temporary catheter use rate after follow-up between the two groups in CKD 4-5**

| **Group** | **Mortality** | **RRT** | | | | **Group** | **Access selection during initial HD** | |
| --- | --- | --- | --- | --- | --- | --- | --- | --- |
|  |  | **Total** | HD | PD | RT |  | CVC | TCC or AVF |
| APP  +Outpatient  N=512 | 20(3.9) | 150(29.2) | 114(76.0) | 25(16.6) | 11(7.3) | APP  +Outpatient  N=114 | 60(52.6) | 54(47.3) |
| Traditional Outpatient  N=499 | 13(2.6) | 153(30.6) | 101(66.0) | 49(32.0) | 3(1.9) | Traditional Outpatient  N=101 | 31(30.6) | 70(69.3) |
| Pearson's chi-squared test | 1.36 | .11 | 13.11 | | | Pearson's chi-squared test | 10.59 | |
| *P* value | .24 | .73 | <.001 | | | *P* value | <.001 | |

Abbreviation: RRT, renal replacement therapy; HD, hemodialysis; PD, peritoneal dialysis; RT, renal transplantation;

CVC, central venous catheter;TCC, tunnel-cuffed catheter;AVF, Arteriovenous Fistula.
